# Metabolic reprogramming induced by CRP deficiency or human CRP transgenic in influenza-infected mice

**DOI:** 10.3389/fimmu.2026.1683431

**Published:** 2026-03-09

**Authors:** Junhao Luo, Zhuohan Zhang, Song Zhao, Siyu Pu, Li Li, Rongbao Gao

**Affiliations:** NHC Key Laboratory of Biosafety, NHC Key Laboratory of Medical Virology and Viral Diseases, Chinese National Influenza Center, National Institute for Viral Disease Control and Prevention, Chinese Center for Disease Control and Prevention, Beijing, China

**Keywords:** C-reactive protein, influenza, glutathione, kynurenine, metabolic reprogramming, metabolomics, vitamin B6

## Abstract

**Introduction:**

C-reactive protein (CRP) plays dual roles in influenza infection, contributing to immune protection but potentially exacerbating severe outcomes.

**Methods:**

Here, we investigated CRP-driven metabolic reprogramming in influenza A (H1N1)-infected mice. Metabolomic profiling was performed on lung tissues from wild-type (WT), CRP-deficient (KO), and human CRP transgenic (KI) mice. Correlations were analyzed between metabolites and immune checkpoint LAIR-1, viral load, and serum IL-17/IFN-γ levels.

**Results:**

In WT mice, H1N1 infection triggered metabolic resource redistribution, dynamic inflammatory regulation, antioxidant responses, and immune cell activation. Conversely, KI mice exhibited impaired PUFA/PLA2-mediated inflammatory control. KO mice showed hypoimmunity with premature tryptophan-kynurenine shift, glutathione and proline synthesis defects, etc. Oxidized glutathione and kynurenine correlated significantly with immune checkpoint LAIR-1, or viral TCID_50_.

**Discussion:**

These findings demonstrate that CRP deficiency or human CRP transgenic induces distinct metabolic reprogramming post-infection. Metabolic alterations, particularly in energy redistribution, antioxidant defense, and immune-related pathways, may serve as biomarkers for disease progression in severe influenza. The results highlighted CRP's role in balancing metabolic and immune homeostasis during viral infection.

## Introduction

Influenza virus infection is one of the most common infectious agents and is responsible for seasonal epidemics, leading to 3–5 million severe infections and 290,000–650,000 associated deaths annually (1). Despite annual vaccination programs, the high mortality rates and various complications (e.g., chronic lung disease, cardiac disease, and metabolic disorders) caused by influenza infection are not yet adequately addressed (2, 3). In addition, the influenza pandemic has greatly increased over the past years, with emergencies of avian influenza viruses (e.g., H5N1, H7N9, and H5N6) occurring in humans in several countries or regions (4, 5). Excessive immune response leading to lung injury plays a key role in the mortality or deterioration of severe influenza (6). However, there are many unresolved challenges in the prevention and treatment of influenza, including the host immune response dynamics, the regulation of tissue damage and its repair, and even the lack of biological indicators for the diagnosis and treatment of severe influenza.

C-reactive protein (CRP), an inflammatory biomarker and immune mediator (7, 8), has been demonstrated to play a critical role in the pathogenesis of severe influenza (9, 10) and may contribute to metabolic disorders in inflammatory diseases (11, 12). Our previous studies have shown that CRP mediating immunopathological lesions may be a potential treatment option for severe influenza (13). However, both CRP deficiency and human CRP transgene treatment adversely affected disease progression after influenza virus infection in mice (14). Previous studies have demonstrated that influenza infection affects a variety of cellular metabolic pathways to ensure an optimal environment for its replication and the production of viral particles, some of which are associated with inflammatory responses (15). However, the relationship between CRP and the metabolic disorder caused by influenza infection is unknown, as well as whether or not metabolism-associated biomarkers can be used to examine the progression of the disease.

In this study, to investigate the metabolic state changes caused by CRP or influenza virus, we determined the metabolomics in the lung tissue of wild-type (WT), human CRP knock-in (KI), and CRP knockout (KO) mice infected with lethal influenza A (H1N1) virus, as well as assessed and compared the metabolic state differences and the correlations between metabolites and the immune response or viral load among the three mouse groups. The results would provide a mechanistic explanation from a metabolic biology perspective for whether CRP deficiency or human CRP transgenic treatment exacerbates severe influenza virus infection in mice and characterize potential disease progression biomarkers or therapeutic targets for severe influenza.

## Materials and methods

### Samples

We used the lung samples from our previous study for the metabolomics detection (14). In brief, 8- to 10-week-old CRP KO, human CRP KI, or WT C57BL/6J female mice were infected by intranasal inoculation of 1.5 × 10^4^ TCID_50_ (50% tissue culture infectious dose) of A/California/04/2009 (H1N1) in 50 μl phosphate-buffered saline (PBS), and mice were anesthetized with 2% isoflurane (0.41 ml/min at 4 L/min fresh gas flow) before infection. The left lung tissues were taken and lyophilized from mice euthanized with CO_2_ (30% chamber volume/min) at 3 or 7 days post-infection (dpi) or mice without infection as mock controls. The sample sizes are as follows: 12 WT mice (four for 0 dpi, four for 3 dpi, and three for 7 dpi), 13 KI mice (four for 0 dpi, four for 3 dpi, and five for 7 dpi), and 12 KO mice (four for 0 dpi, three for 3 dpi, and five for 7 dpi). All animal studies were performed according to the guidelines approved by the Investigational Animal Care and Use Committee of the National Institute for Viral Diseases Control and Prevention of the China CDC and were conducted following the guidelines of the Council for Animal Care.

### Sample processing

We picked ~5 mg of lyophilized lung sample for each mouse into a 2-ml centrifuge tube with 1 ml of methanol and 20 μl of the internal standard substance (myristic acid-1,2–^13^C_2_, 300 μg/ml) and vortexed for 30s. Subsequently, the samples were sonicated for 32 s, incubated in an ice water bath for 20 min, and centrifuged at 13,000 rpm for 5 min at 4°C. From each sample, 800 μl of supernatant was obtained and nitrogen-dried. The samples were resolved with 100 μl of acetonitrile/water (50:50, *v*/*v*). After centrifugation at 13,000 rpm for 5 min, 60 μl of the supernatant was used for LC-MS detection.

### QC sample preparation

Equal volumes were taken from each sample prepared for LC-MS detection in the previous step and mixed to form one large sample, which was then divided into nine quality control (QC) samples for instrument precision and stability monitoring. To assess instrument precision, three QC samples were run at the start of the test. Thereafter, for every six samples tested, one QC sample was run to monitor the stability of the instrument (**Supplementary Figure 1**).

### Liquid chromatography parameters

In the positive ion mode, the mobile phase was 0.1% formic acid in water (liquid A) and 0.1% formic acid in 100% methanol (liquid B). In the negative ion mode, the mobile phase was 10 mM ammonium formate in water (liquid A) and 10 mM ammonium in 95% methanol formate (liquid B). The chromatographic gradient is shown in **Supplementary Table 1**, with a flow rate of 0.3 ml/min, column temperature of 35°C, and an injection volume of 2 μl.

### Mass spectrometry parameters

The scanning mode scanned the positive and negative ions separately. The detection mode was full mass/data-dependent tandem mass spectrometry (dd-MS2), with resolutions of 70,000 (full mass) and 17,500 (dd-MS2). The electrospray voltage values were 3.8 kV (positive) and 3.2 kV (negative). The capillary temperature was 300°C, the sheath gas flow rate was 40 AU, and the nebulizer temperature was 350°C.

### Metabolite data processing and analysis

The three-dimensional (3D) matrix dataset consisting of the names, groups, and peak heights of the metabolites was extracted from the LC-MS spectra using the software MS-DIAL (16) and the MassBank database for peak extraction and substance identification. After total ion flow normalization, log transformation, and Pareto scaling of the data, the data generally conformed to the bell-shaped distribution (*Supplementary material 2*). Subsequently, the 3D matrix dataset was imported into SIMCA software for principal component analysis (PCA) and partial least squares discriminant analysis (PLS-DA) and was screened for differential metabolites with variance inflation factor (VIP) >1.0 (17). The data of pre-infection (0 dpi) and of 3 or 7 dpi in the same group of mice were subjected to the fold change (FC) test, and a FC value >1.5 (higher metabolite level than before infection) or <0.667 (lower metabolite level than before infection) (18) was used to determine differential substances between the two groups. Finally, the Benjamini–Hochberg false discovery rate (BH-FDR)-corrected one-way ANOVA was used to screen the statistically significant differential metabolites between pre-infection mice and mice with different infection durations according to a *q*-value <0.05. The intersection of the metabolites obtained from the VIP > 1.0, FC > 1.5 (or <0.667), and *q*-value < 0.05 screening were selected as the signature differential metabolite. Data were analyzed for metabolic pathway enrichment in MetaboAnalyst (http://www.metaboanalyst.ca/).

### Statistical analysis

To determine the potential connection between the changes in metabolites and the immune response, we analyzed correlations between the differential metabolite signatures and the checkpoint levels in our previous study (14). The correlations of all detected metabolites with the relative expression levels of six immune checkpoint mRNAs (i.e., *GITR*, *BTLA*, *TIM-3*, *CTLA-4*, *LAIR-1*, and *PD-1*), the levels of interleukin 17 (IL-17) and interferon gamma (IFN-γ), and the TCID_50_ of HIN1 in the lung tissues of mice were examined using Pearson’s product-moment correlation analysis [*p < 0.05, **p < 0.01, ***p < 0.0 01 (two-tailed)].

## Results

### LC-MS quality control and analysis results

The total ion chromatograms (TICs) of all QC samples were superimposed (**Supplementary Figure 3A**), which showed good spectral overlap and minimal fluctuations in retention time and peak signal intensity, indicating that the instrument maintained a stable condition throughout the sample detection and analysis. The extracted ion chromatogram (XIC) of the component with a mass-to-charge ratio of 179.0547 in the primary mass spectrum (MS1) was randomly selected and superimposed on the XIC of all the samples (**Supplementary Figure 3B**). The retention time of the same component in the different samples was consistent with a good peak shape, indicating that the quantitative results of this experimental method were accurate and that the data were reliable.

### Metabolite profiling and differential metabolite identification

**Figure 1:**
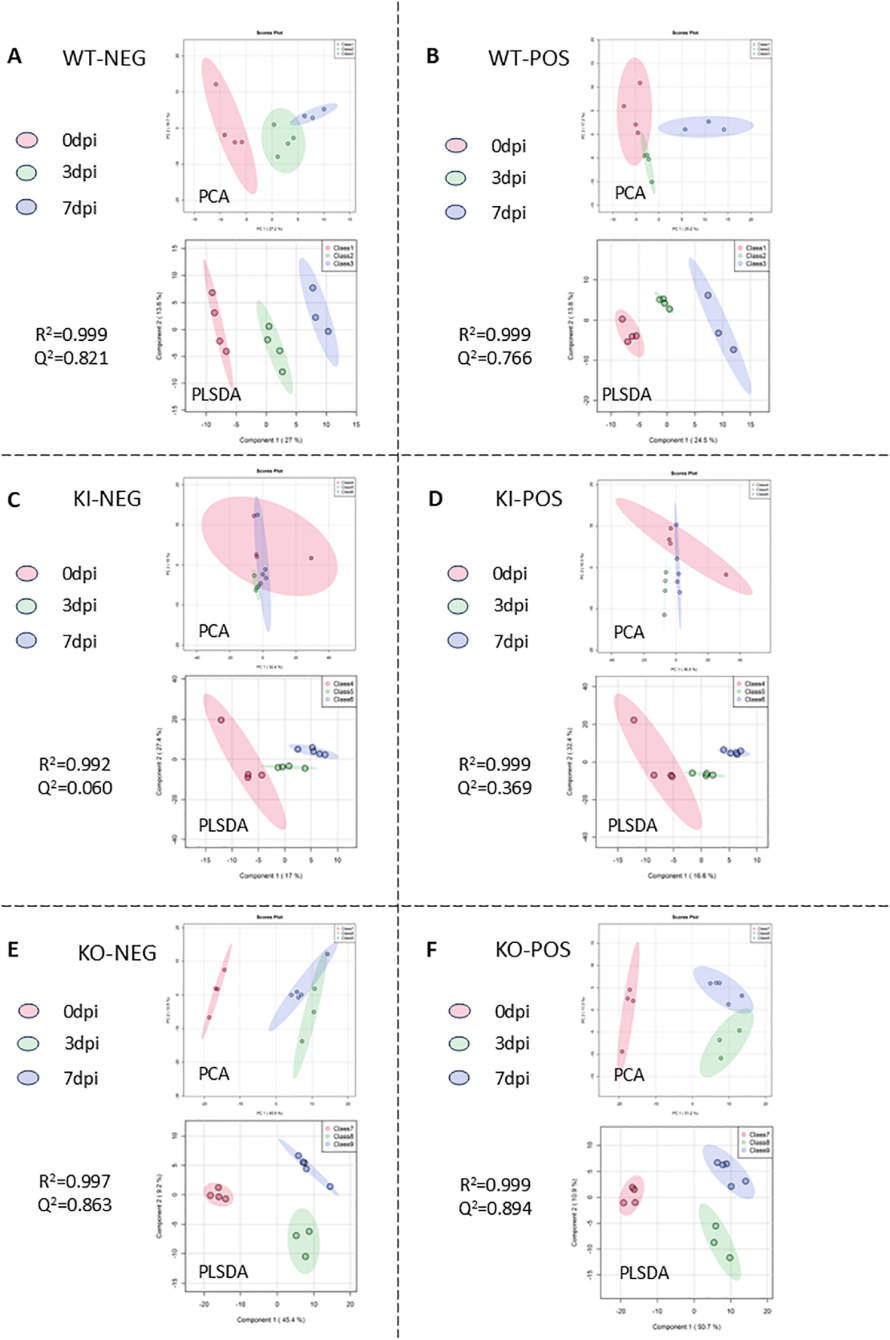
Dimensionality reduction of metabolite data. Data were normalized and Log10-transformed using the Pareto scaling method, and the corresponding score plot was obtained with principal component analysis (PCA) and partial least squares discriminant analysis (PLS-DA), with data points of *different colors* representing different groups. **(A, B)** PCA and PLS-DA score plots of wild-type (WT) mice in negative **(A)** and positive **(B)** ion mode metabolites before infection (class 1, *red*), at 3 days post-infection (dpi) (class 2, *green*), and at 7 dpi (class 3, *blue*). **(C, D)** PCA and PLS-DA score plot of human C-reactive protein (CRP) knock-in (KI) mice in negative **(C)** and positive **(D)** ion mode metabolites before infection (class 4, *red*), at 3 dpi (class 5, *green*), and at 7 dpi (class 6, *blue*). **(E, F)** PCA and PLS-DA score plots of CRP knockout (KO) mice in negative **(E)** and positive **(F)** ion mode metabolites before infection (class 7, *red*), at 3 dpi (class 8, *green*), and at 7 dpi (class 9, *blue*).

Given the limited sample availability, some experimental groups had only *n* = 3 mice (WT at 7 dpi and KO at 3 dpi). While all reported differential metabolites met stringent statistical criteria including FDR correction, the findings from these smaller groups should be interpreted with appropriate caution and would benefit from independent validation. A total of 379 compounds were identified, which included a variety of amino acids, fatty acids, purines, pyrimidines, and other organic compounds. These are widely distributed in the pathways of energy and immune metabolism. The metabolite information obtained after MS-DIAL processing was imported into SIMICA 13.0 for PCA and PLS-DA and plotted in a score plot (**Figure 1**). The metabolite profiles of each group of samples could be observed in the PCA score plot, with each point in the plot representing one sample and all points being distributed in the ellipse area (95% confidence interval). Of these, the *R*^2^ values of the PLS-DA score plots were all greater than 0.95, indicating that the differentiation and the accuracy of the mathematical model were statistically significant. This suggests that the metabolites in the lung tissues of mice at 3 dpi or at 7 dpi in each group were significantly different from those without infection. The VIP values of each variable in the lung tissue samples were calculated according to the PLS-DA model.

According to VIP > 1.0, FC > 1.5 (or <0.667), and BH-FDR-corrected one-way ANOVA *q*-value <0.05, the metabolites with significant changes in the lung tissues of each group of mice at 3 or 7 dpi compared with the lung tissues of mice without infection were obtained. The results from both negative and positive ion modes for each type of mouse were combined to generate a list of differential metabolites for each mouse type before and after infection (**Supplementary Table 2**). Of the differential metabolites, 58 were identified in WT mice, 36 were identified in KI mice, and 94 were identified in KO mice.

### Post-infection metabolic change patterns in mice

**Figure 2:**
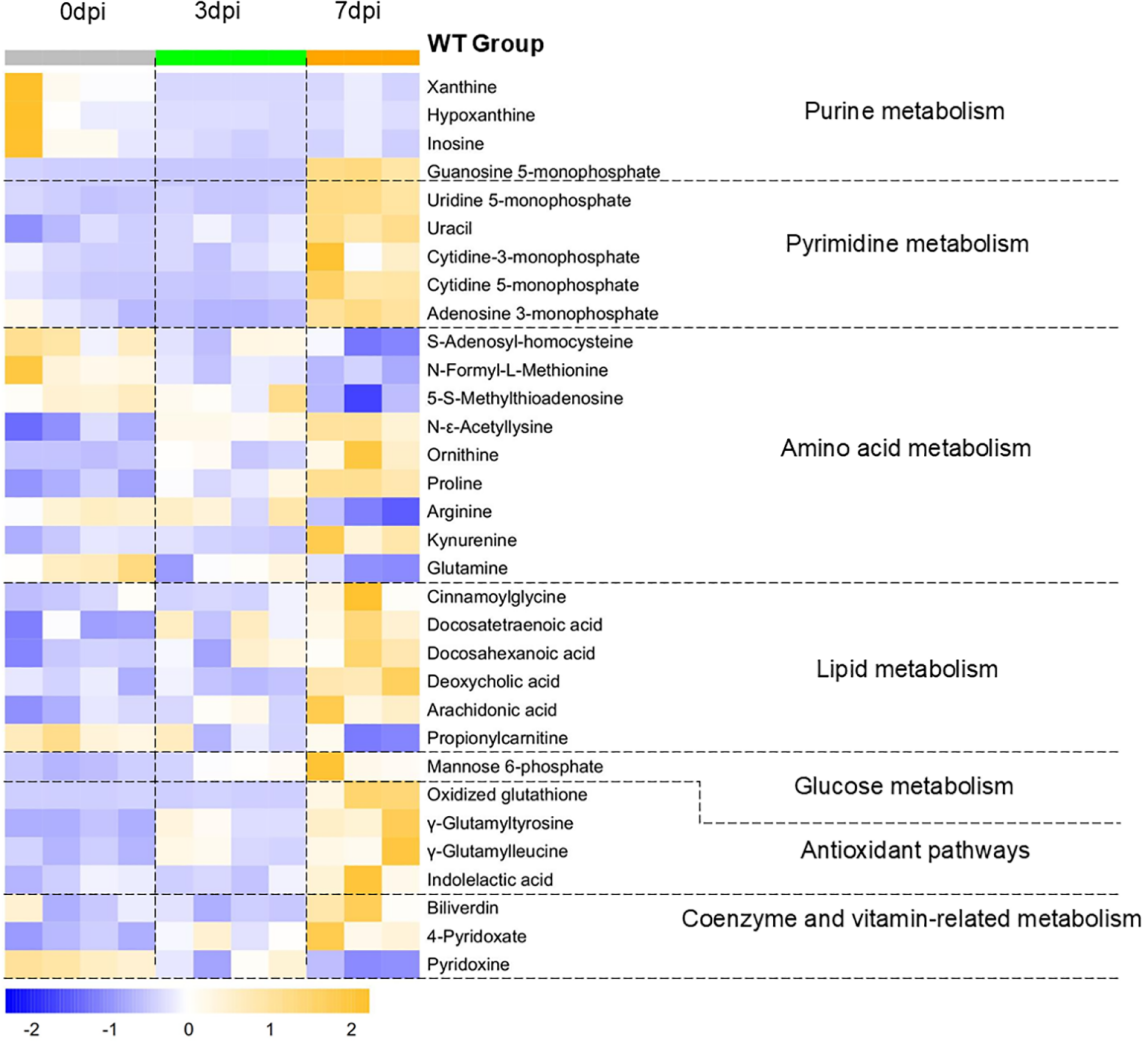
Changes in the pulmonary differential metabolites in wild-type (WT) mice at 3 days post-infection (dpi) and 7 dpi. Differential metabolites in WT mice at 3 and 7 dpi compared with 0 dpi were screened using the criteria variance inflation factor (VIP) >1.0, fold change (FC) >1.5 or <0.667, and Benjamini–Hochberg false discovery rate (FDR)-corrected one-way ANOVA *q*-value <0.05. Normalized metabolite quantity data are visualized as a heatmap, with *orange* indicating higher metabolite levels and *blue* indicating lower levels. Samples of 0 dpi are labeled in *gray*, 3 dpi in *green*, and 7 dpi in *orange*.

**Figure 2** shows the main pulmonary differential metabolites between WT mice without infection and mice with infection at 3 and 7 dpi. Differential metabolites whose biological significance can be clearly annotated are listed in **Table 1** according to their associated metabolic pathways. The sustained decrease of xanthine, hypoxanthine, and inosine and the early-stage decrease of guanosine 5-monophosphate in purine metabolism, as well as the early-stage decrease of uridine 5-monophosphate and cytidine-3-monophosphate in pyrimidine metabolism, indicated that the virus exploited the host’s energy metabolism to support its own replication. The progressive increase of *N*-epsilon-acetyllysine, mannose 6-phosphate (M6P), and cinnamoylglycine indicated that the virus counteracted the antiviral effects of the immune system by interfering with the host immune metabolism. The progressive decrease of propionylcarnitine, *S*-adenosyl-homocysteine, and *N*-formyl-l-methionine in methionine metabolism and the β-oxidation of fatty acid indicated that the host’s immune response and antiviral effects gradually intensified as the infection progressed. The progressive changes of polyunsaturated fatty acid (PUFA) metabolism, phospholipase A2 (PLA2) pathway, glutamate metabolism, vitamin B6 metabolism, and the later-stage changes of tryptophan (Trp) metabolism, heme metabolism, and bile acid metabolism indicated that the host’s dynamic regulation of the pro-inflammatory and anti-inflammatory responses, antioxidant system, and immune balance regulation started to exert their respective effects as the infection progressed. The later-stage upregulation of guanosine 5-monophosphate and pyrimidine metabolism and the progressive increase of ornithine and proline reflected the recovery of host energy metabolism and the initiation of tissue damage repair. In summary, the metabolic state of WT mice was characterized by viruses exploiting the host energy resources while disrupting the immune metabolism processes, as well as the host’s antiviral effects and its attempts to restore homeostasis.

**Table 1:** Post-infection metabolic change patterns in wild-type (WT) mice.

Metabolite name	Pathway	Change patterns	Biological significance
Xanthine	Purine metabolism	↓ Since 3 dpi	This may suggest that the virus persistently exploited purine metabolism and exacerbated the energy crisis in host cells, with the salvage synthesis pathway for RNA repair remaining persistently activated (19–21).
Hypoxanthine		↓ Since 3 dpi	
Inosine		↓ Since 3 dpi	
Guanosine 5-monophosphate		3 dpi↓, 7 dpi↑	During the transcription initiation phase, the virus relied on the guanosine cap structure (22). The change patterns of guanosine 5'-monophosphate (GMP) may suggest that, during the early stage of infection, the virus exploited the host’s guanosine triphosphate (GTP) catabolism. However, this phenomenon disappeared during the mid-to-late stages of infection, followed by a rise in the GMP levels.
Uridine 5-monophosphate	Pyrimidine metabolism	7 dpi↑	The levels of the pyrimidine nucleotide precursors initially decreased and then increased, which may suggest that the host’s energy metabolism strategy shifted from glycolysis to nucleotide synthesis. This may further indicate a transition from immune consumption–metabolic suppression to tissue repair–metabolic reconstruction (20, 23, 24).
Uracil		7 dpi↑	
Cytidine-3-monophosphate		7 dpi↑	
Cytidine 5-monophosphate		7 dpi↑	
Adenosine 3-monophosphate		3 dpi↓, 7 dpi↑	
Arginine	Arginine and proline metabolism	7 dpi↓	The urea cycle was activated, and proline synthesis was continuously upregulated to support collagen synthesis and antioxidation, which may suggest dynamic regulation of pulmonary tissue repair and inflammation (31, 32).
Ornithine		↑ Since 3 dpi	
Proline		↑ Since 3 dpi	
Kynurenine	Tryptophan metabolism	7 dpi↑	This may suggest that tryptophan metabolism shifted toward aryl hydrocarbon receptor (AhR) activation-mediated immunosuppressive direction (25).
Indolelactic acid		7 dpi↑	Indolelactic acid is a gut-derived tryptophan metabolite that exhibit antioxidant activity (47).
*S*-Adenosyl-homocysteine (SAH)	Methionine metabolism	7 dpi↓	Alterations in the methylation levels could influence the expression of immune-related genes. For example, DNA hypomethylation could promote the transcription of pro-inflammatory cytokines (e.g., IL-6 and TNF-α)). In the methionine cycle, the methyl donor *S*-adenosylmethionine (SAM) generated SAH and MAT after completing the methylation reaction and was subsequently recycled back into SAM through a series of enzymatic reactions. The time-dependent downregulation enhancement of SAH and MAT may suggest that, as the infection progressed, the methionine cycle was accelerated to exert antiviral effects (48, 49).
5-S-Methylthioadenosine (MAT)		7dpi↓	
*N*-formyl-l-methionine (fMet)		↓ Since 3 dpi	fMet participated in the posttranslational modification process of mitochondrial protein synthesis, and the time-dependent enhancement of its consumption likely reflected the enhanced synthesis of the antiviral-related mitochondrial proteins such as MAVS as the infection progressed (50).
*N*-epsilon-acetyllysine	Lysine metabolism	↑ Since 3 dpi	Histone acetylation was enhanced, which could promote the expression of inflammatory genes, but could also be utilized by the virus to negatively regulate the antiviral response (26).
Docosatetraenoic acid (DTA)	PUFA metabolism	7 dpi↑	DTA is an *ω*-6 fatty acid derivative that exhibits relatively weak anti-inflammatory activity (33).
Docosahexaenoic acid (DHA)		7 dpi↑	DHA is a potent anti-inflammatory mediator (34).
Deoxycholic acid (DCA)	Bile acid metabolism	7 dpi↑	DCA is produced by the gut microbiota through metabolism and alleviates inflammatory response and immune injury (35).
Arachidonic acid (ARA)	PLA2 pathway	7 dpi↑	ARA is a substrate of COX/LOX and serves as a precursor of pro-inflammatory mediators (34).
Propionylcarnitine	β-oxidation of fatty acid	7 dpi↓	The time-dependent enhancement of fatty acid β-oxidation downregulation may suggest that the host might redirect the metabolic resources to glycolysis in order to support the rapid proliferation of immune cells during the early stage of infection (15). With the persistent oxidative stress and inflammatory response, propionylcarnitine was further consumed as an antioxidant (40).
Glutamine	Glutamate metabolism	↓ Since 3 dpi	Glutamine, as the precursor compound for GSH synthesis, exhibited a time-dependent enhancement of its depletion, and the level of the antioxidative reaction product, oxidized GSH, was increased, which may suggest exacerbated oxidative stress and the mobilization of the antioxidative reserves (36).
Oxidized glutathione (oxidized GSH)		7 dpi↑	
Biliverdin	Heme metabolism	7 dpi↑	Biliverdin, as a product of HO-1 activation, may suggest that heme metabolism might have been upregulated and involved in antioxidation (51).
Mannose 6-phosphate (M6P)	M6P pathway	↑ Since 3 dpi	The synthesis of M6P-dependent cathepsins exhibited a time-dependent enhancement, which may facilitate the viral suppression of antigen presentation and immune escape (28–30).
4-Pyridoxate (PA)	Vitamin B6 metabolism	↑ Since 3 dpi	Pyridoxal-5'-phosphate (PLP) serves as the active coenzyme form of vitamin B6, with PN being its precursor and PA the terminal product derived from PLP metabolism. The changes in their levels may indicate a time-dependent enhancement of vitamin B6 metabolism, suggesting that the host’s demand for the coenzyme increased to exert anti-inflammatory and immunomodulatory effects (37).
Pyridoxine (PN)		7 dpi↓	
Cinnamoylglycine	Glycine metabolism	7 dpi↑	The increased cinnamoylglycine levels may suggest enhanced conjugation of glycine with cinnamic acid, which might affect the active glycine levels in pulmonary tissues and weaken the anti-inflammatory effects of glycine (27), potentially associated with post-infection metabolic reprogramming.

*PUFA*, polyunsaturated fatty acid; *dpi*, days post-infection; *PLA2*, phospholipase A2.

This table lists the differential metabolites that can be clearly annotated with biological significance in the lungs of wild-type (WT) mice at 3 and 7 dpi and summarizes the metabolites belonging to the same metabolic pathway through enrichment analysis. “7dpi ↓ or ↑”/”7dpi↓/↑” denote later-stage change, where the metabolite levels showed no significant change at 3 dpi but decreased or increased at 7 dpi. “3dpi↓,7dpi↑” denotes a metabolite level decreased at 3 dpi and increased at 7 dpi. “↓ or ↑ since 3 dpi” denotes a metabolite level decreased or increased at 3 dpi and sustained to 7 dpi.

**Figure 3:**
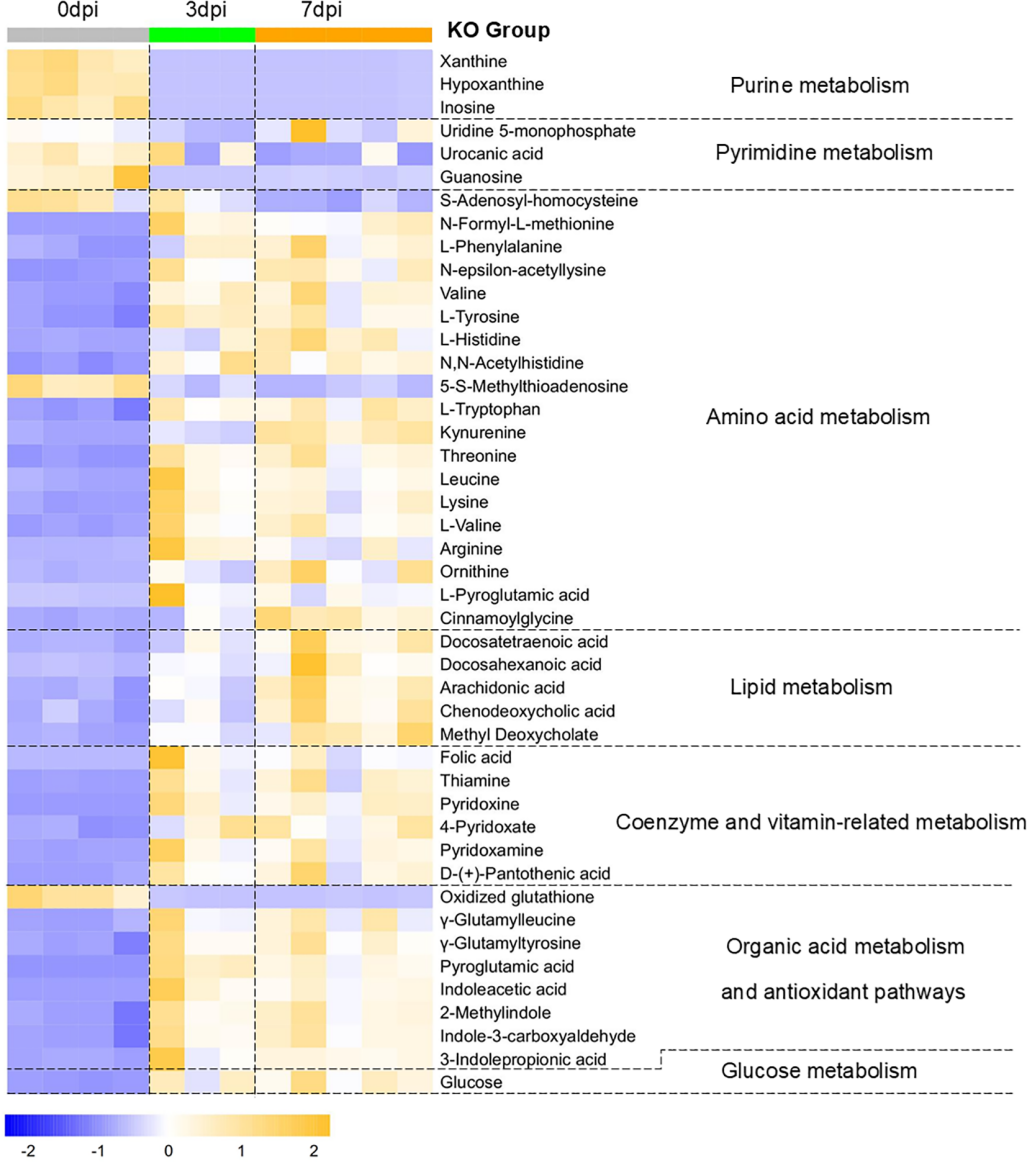
Changes in the pulmonary differential metabolites in KO mice at 3dpi and 7dpi. Differential metabolites in C-reactive protein (CRP) knockout (KO) mice at 3 and 7 days post-infection (dpi) compared with 0 dpi were screened using the criteria of variance inflation factor (VIP) >1.0, fold change (FC) >1.5 or <0.667, and Benjamini–Hochberg false discovery rate (FDR)-corrected one-way ANOVA *q*-value <0.05. Normalized metabolite quantity data were visualized as a heatmap, with *orange* indicating higher metabolite levels and *blue* indicating lower levels. Samples of 0 dpi are labeled in *gray*, 3 dpi in *green*, and 7 dpi in *orange*.

**Figure 4:**
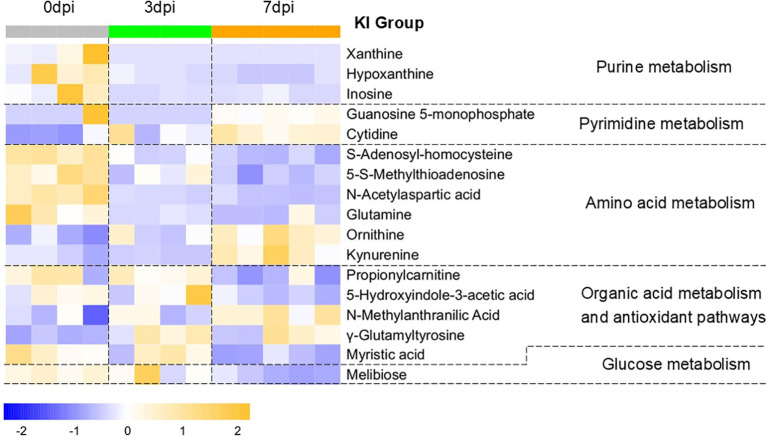
Changes in the pulmonary differential metabolites in human C-reactive protein (CRP) knock-in (KI) mice at 3 days post-infection (dpi) and at 7 dpi. Differential metabolites in KI mice at 3 and 7 dpi compared to 0 dpi were screened using the criteria of variance inflation factor (VIP) >1.0, fold change (FC) >1.5 or <0.667, and Benjamini–Hochberg false discovery rate (FDR)-corrected one-way ANOVA *q*-value < 0.05. Normalized metabolite quantity data are visualized as a heatmap, with *orange* indicating higher metabolite levels and *blue* indicating lower levels. Samples of 0 dpi are labeled in *gray*, 3 dpi in *green*, and 7 dpi in *orange*.

Using the metabolic change patterns of WT mice with lethal H1N1 infection as a reference, we analyzed the differences in the metabolic features (i.e., temporal dynamics, magnitude of changes, and upward/downward trends) between KO and KI mice with H1N1 infection and annotated the effects of CRP knockout or human CRP knock-in on the host energy and immune metabolism. **Figures 3**, **4** present the changes in the main pulmonary differential metabolites in KO and KI mice at 3 and 7 dpi, respectively. The comparable metabolic patterns between KI/KO mice and WT mice in lung tissues are summarized in **Table 2**. For KI/KO mice, the alterations in the metabolic patterns can be categorized into two types: one that may represent adverse effects on infection control attributed to CRP deficiency or human CRP transgenic treatment and another that may reflect the host compensatory responses that emerge under conditions of abnormal CRP expression.

**Table 2:** Comparison of the post-infection metabolic change patterns among wild-type (WT) and C-reactive protein (CRP) knock-in (KI) and knockout (KO) mice.

Metabolite name	Pathway	Change patterns			Biological significance
		WT	KI	KO	
Xanthine	Purine metabolism	↓ Since 3 dpi			The purine metabolism in WT, KI, and KO mice was synchronously downregulated post-infection, which may reflect the viruses’ non-selective exploit of purine metabolism across all three groups (19–21).
Hypoxanthine					
Inosine					
Ornithine	Arginine and proline metabolism	↑ Since 3 dpi	↑ Since 3 dpi	↑ Since 3 dpi	The increased proline synthesis in WT mice may suggest the host’s attempt at lung tissue repair, while KI/KO mice exhibited proline synthesis dysfunction, which may have contributed to the impaired tissue damage repair and antioxidant defense (31, 32).
Proline		↑ Since 3 dpi	–	–	
Arginine		7 dpi↓	–	↑ Since 3 dpi	
l-Tryptophan	Tryptophan metabolism	–	–	↑ Since 3 dpi	The uptake of tryptophan in the lung tissues of KO mice was enhanced, and tryptophan metabolism shifted earlier toward an immunosuppressive direction, which may suggest that CRP deficiency is associated with a low immune response state (25).
Kynurenine		7 dpi↑		↑ Since 3 dpi	
Indolelactic acid		7 dpi↑	–	↑ Since 3 dpi	The levels of indole derivatives in KO mice increased, which might be associated with gut microbiota metabolic reprogramming, suggesting potential compensatory mechanisms following the collapse of the host antioxidant system (38, 42).
3-Indolepropionic acid		–	–	↑ Since 3 dpi	
5-Hydroxyindole-3-acetic acid (5-HIAA)		–	7 dpi↓	–	In the late stage of infection, the tryptophan metabolism in KI mice shifted toward kynurenine synthesis, while serotonin synthesis was downregulated (5-HIAA is the ultimate metabolic end-product of serotonin) to enhance immunosuppression, which may have served as a host response mechanism to counteract excessive immune activation associated with CRP overexpression (38).
*N*-methylanthranilic acid (NMA)		–	7 dpi↑	–	The increased NMA levels in KI mice were associated with gut microbiota metabolic reprogramming, which may have served as the host’s antioxidant bypass mechanism to counteract the excessive immune activation associated with CRP overexpression (38).
Leucine	Amino acid metabolism	–	–	↑ Since 3 dpi	KO mice might upregulate the synthesis or uptake of multiple amino acids related to energy metabolism or immune function, which might partially compensate for the immunosuppression associated with CRP deficiency (52–54).
Lysine		–	–	↑ Since 3 dpi	
l-Valine		–	–	↑ Since 3 dpi	
Tyrosine		–	–	↑ Since 3 dpi	
Docosatetraenoic acid (DTA)	PUFA metabolism	7 dpi↑	–	↑ Since 3 dpi	KO mice might utilize enhanced PUFA/PLA2 metabolism to promote inflammatory responses and synthesize anti-inflammatory mediators, which might partially compensate for the immunosuppression and the absence of the antioxidant system associated with CRP deficiency. The lack of dynamic pro-inflammatory/anti-inflammatory regulation in KI mice may reflect the immune regulatory imbalance associated with CRP overexpression (33, 34).
Docosahexaenoic acid (DHA)		7 dpi↑	–	↑ Since 3 dpi	
Arachidonic acid (ARA)	PLA2 pathway	7 dpi↑	–	↑ Since 3 dpi	
Propionylcarnitine	β-oxidation of fatty acid	7 dpi↓	7 dpi↓	–	WT and KI mice exhibited downregulation of β-oxidation of fatty acids as early as the infection progresses, which may reflect metabolic reprogramming and immune activation, while the absence of regulation in KO mice may reflect the low immune response state associated with CRP deficiency (15, 40).
Myristic acid	Fatty acid metabolism	–	7 dpi↓	–	Myristic acid, acting as a metabolic checkpoint of the innate immune response, was decreased in KI mice during the late stage of infection, which may suggest that CRP overexpression is associated with immune dysregulation (39).
Glutamine	Glutamate metabolism and γ-glutamyl cycle	7 dpi↓	↓ Since 3 dpi	–	WT mice exhibited intensified oxidative stress, prompting the activation of antioxidant defenses, with upregulated GSH synthesis manifested as increased levels of oxidized GSH. In contrast, KO mice showed elevated levels of γ-glutamyl cycle-associated metabolites, which may suggest an impaired GSH synthesis and a collapse of the antioxidant system, which was reflected by decreased levels of oxidized GSH (36).
Oxidized glutathione (oxidized GSH)		7 dpi↑	–	3 dpi↓, 7 dpi⇊	
l-Pyroglutamic acid		–	–	↑since 3dpi -	
γ-Glutamylleucine		7 dpi↑	–	↑since 3dpi -	
γ-Glutamyltyrosine		↑ Since 3 dpi–	↑ Since 3 dpi	↑ Since 3 dpi–	
Cytidine	Methionine metabolism	–	↑ Since 3 dpi–	–	The methionine cycle showed similar patterns in WT and KI mice. However, in KO mice, the accumulation of metabolites such as methionine, l-histidine, and folic acid may suggest that the methionine cycle was impaired, which might be associated with a decreased production of cysteine (a substrate for GSH synthesis) and a failed GSH synthesis (41).
*S*-Adenosyl-homocysteine		7 dpi↓	↓ Since 3 dpi	–	
5-*S*-Methylthioadenosine		7 dpi↓	↓ Since 3 dpi	–	
Methionine		–	–	↑ Since 3 dpi	
l-Histidine		–	–	↑ Since 3 dpi	
Folic acid		–	–	↑Since 3 dpi	
Deoxycholic acid	Bile acid metabolism	7 dpi↑	–	–	WT mice might achieve multi-pathway anti-inflammatory and antioxidant effects during the late stage of infection through an enhanced synthesis of biliverdin and enhancement of the gut–lung metabolic axis of bile acids (35, 51).. The absence of these pathways in KO and KI mice may suggest that immune homeostasis dysregulation and gut microbiota dysbiosis occurred.
Biliverdin	Heme metabolism	7 dpi↑	–	–	
4-Pyridoxate (PA)	Vitamin B6 metabolism	↑ Since 3 dpi	–	↑ Since 3 dpi	WT mice exhibited upregulated vitamin B6 metabolism, which may suggest that the host exerted anti-inflammatory and immunomodulatory effects through the coenzyme function of PLP. In KO mice, the increased uptake of PM by the lung tissue and the earlier upregulation of vitamin B6 metabolism might have been a compensatory pathway due to the absence of the host antioxidant system. The absence of vitamin B6 metabolism in KI mice may reflect immune homeostasis dysregulation associated with CRP overexpression (37).
Pyridoxine (PN)		7 dpi↓	–	↑ Since 3 dpi	
Pyridoxamine (PM)		–	–	↑ Since 3 dpi	

*PUFA*, polyunsaturated fatty acid; *dpi*, days post-infection; *PLA2*, phospholipase A2.

This table lists differential metabolites that can be clearly annotated with biological significance in the lungs of KI and KO mice at 3 and 7 dpi, along with their comparative changes relative to WT mice. Metabolites belonging to the same metabolic pathway were grouped together through enrichment analysis. “↓ or ↑ since 3 dpi” denotes a metabolite level decreased or increased at 3 dpi and sustained to 7 dpi. “7 dpi↓ or ↑” denotes later-stage change, where the metabolite levels showed no significant change at 3 dpi, but decreased or increased at 7 dpi. En dash denotes no significant change detected in a metabolite level before and after infection.

For KI mice, on the one hand, the levels of docosatetraenoic acid (DTA), docosahexaenoic acid (DHA), and arachidonic acid (ARA) in PUFA metabolism and PLA2 pathway and those of 4-pyridoxate (PA)/pyridoxine (PN)/pyridoxamine (PM) in vitamin B6 metabolism did not significantly change during infection, suggesting the absence of PUFA/PLA-mediated dynamic inflammatory regulation mechanisms and vitamin B6-mediated immunomodulatory effects. On the other hand, the later-stage regulation of 5-hydroxyindole-3-acetic acid and *N*-methylanthranilic acid in Trp metabolism and of myristic acid in fatty acid metabolism suggested that KI mice may exhibit certain compensatory effects in response to immune dysregulation.

For KO mice, the level of Trp sustainedly increased and that of kynurenine (Kyn) progressively increased, suggesting that the Trp metabolism shifted prematurely toward an immunosuppressive direction. The levels of proline did not change significantly, while the levels of ornithine and arginine sustainedly increased, suggesting the impairment of tissue damage repair. The level of glutamine did not change significantly, the level of oxidized glutathione (GSH) progressively decreased (was opposite to the pattern in WT mice), and the level of the γ-glutamyl cycle-associated metabolites sustainedly increased, suggesting a collapse of the antioxidant system owing to impaired GSH synthesis. The level of propionylcarnitine in fatty acid β-oxidation did not change significantly, suggesting impairment of the metabolic resource redistribution. On the other hand, the levels of DTA, DHA, and ARA in PUFA metabolism and the PLA2 pathway and those of indoleacetic acid and 3-indolepropionic acid in Trp metabolism sustainedly increased, while vitamin B6 metabolism was sustainedly upregulated, suggesting that KO mice may exhibit certain compensatory effects in response to the impairment of the host antioxidant system. The levels of leucine, lysine, valine, and tyrosine sustainedly increased, suggesting that KO mice may exhibit certain compensatory effects in response to immunosuppression.

In addition, changes in the levels of some of the gut microbiota-derived metabolites, such as indolelactic acid, deoxycholic acid, and bile acid in WT mice, 5-hydroxyindole-3-acetic acid (5-HIAA) and *N*-methylanthranilic acid (NMA) in KI mice, and indole derivatives (indoleacetic acid and 3-indolepropionic acid) in KO mice, were detected in the lungs, suggesting that the lung and the gut may be interconnected through the gut–lung metabolic axis, functioning as an integrated system to participate in host immune regulation following infection.

### Correlation between reprogramming metabolites and the immune response or viral replication in mice

**Figure 5:**
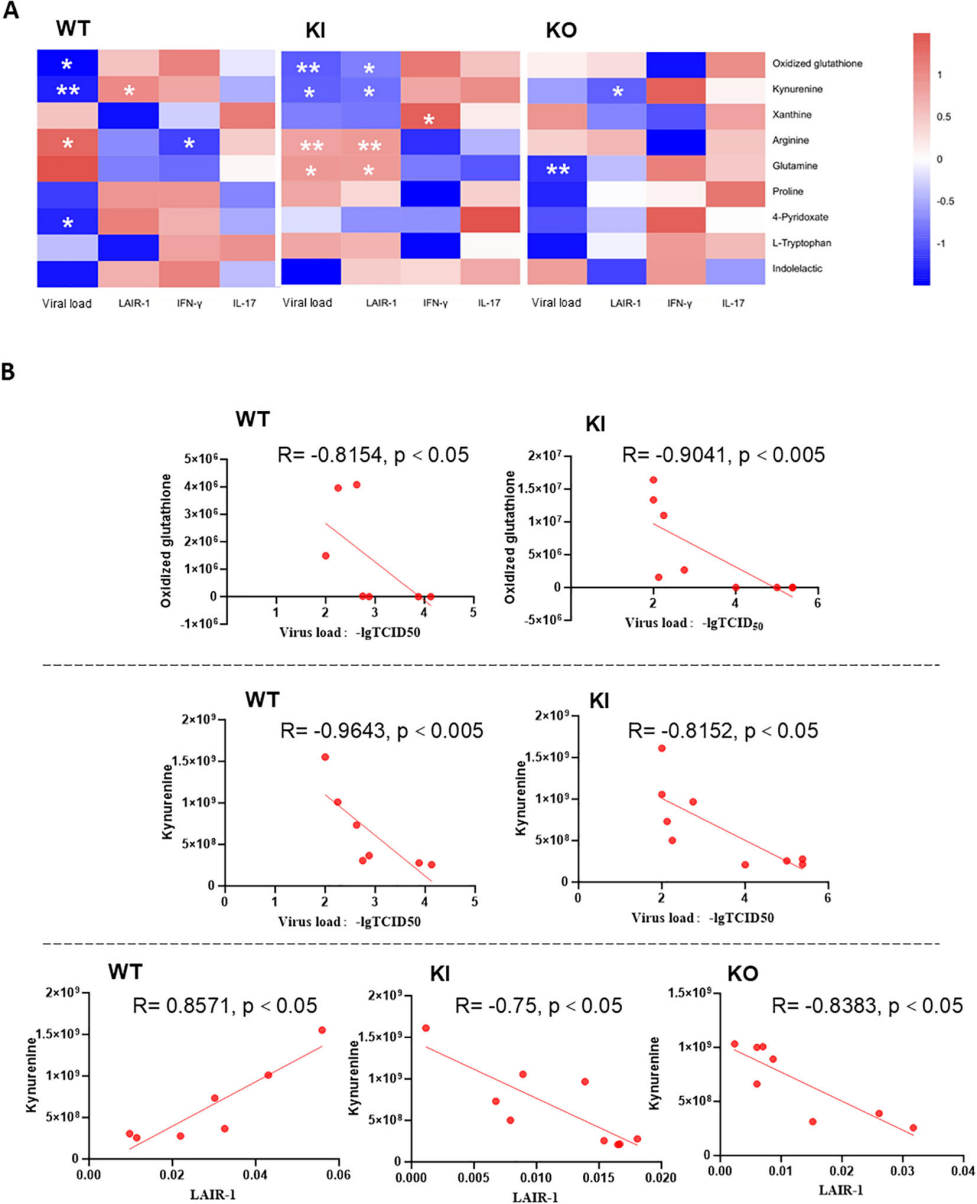
Correlation analysis between metabolites and checkpoints, cytokines, or viral load. **(A)** Heatmap displaying the correlation between C-reactive protein (CRP)-related reprogramming metabolites and the immune checkpoint LIAR-1, viral load, in the lungs, or IL-17 or IFN-γ in sera in wild type (WT), human CRP knock-in (KI), and CRP knockout (KO) mice with influenza A H1N1 infection. Spearman’s correlation analysis was performed. *Red* indicates positive correlation, while *blue* denotes negative correlation. *p < 0.05, **p < 0.005 (two-tailed). **(B)** Scatter plots showing the correlation between oxidized glutathione or kynurenine and viral load in WT and KI mice, as well as the correlation between kynurenine and LAIR-1 in WT, KI, and KO mice.

To determine the potential relationship between reprogramming metabolites and the innate immune response, we examined the correlations of all the main differential metabolites with the mRNA levels of the immune checkpoint LAIR-1, the viral load in the lung tissues, and the levels of IL-17 and IFN-γ in sera using Spearman’s correlation analysis (**Supplementary Figure 5**). The results of some important differential metabolites (e.g., oxidized GSH, Kyn, xanthine, arginine, glutamine, proline, PA, Trp, and indolelactic acid) are displayed in **Figure 5**. The results showed that, in the WT group, viral load was negatively correlated with oxidized GSH, Kyn and PA and positively correlated with arginine. LAIR-1 was positively correlated with Kyn, while IFN-γ was negatively correlated with arginine. In the KI group, viral load and LAIR-1 were negatively correlated with oxidized GSH and Kyn, respectively, and positively correlated with arginine and glutamine, while IFN-γ was positively correlated with xanthine. In the KO group, viral load was negatively correlated with glutamine, while LAIR-1 was negatively correlated with Kyn. There was no significant correlation between IL-17 and the aforementioned metabolites.

## Discussion

CRP, an acute-phase response protein, is generally recognized as a nonspecific inflammatory marker. Our previous studies demonstrated that CRP mediated multiple immunopathological events following severe influenza A infection (13). However, viral challenge experiments revealed that both CRP deficiency and overexpression exacerbated influenza virus infection (14). Previous studies also suggested that CRP may contribute to metabolic disorders in inflammatory diseases. To explore the pathogenesis associated with CRP in severe influenza, in this study, we determined and analyzed the metabolomes of the lung tissues of WT, KO, and KI mice infected with influenza A H1N1 virus.

Our results showed that the metabolic state of WT mice with H1N1 infection exhibited the following two possible characteristics. One was viral exploit of the host energy and disruption of the host immune metabolism, which were demonstrated by exploiting the purine and pyrimidine metabolism to acquire energy and transcriptional components to support early viral replication (19–24); exploiting the Trp metabolism and histone acetylation to enhance immunosuppression and promote viral replication (25, 26); disrupting the glycine metabolism to counteract the host anti-inflammatory effects, leading to tissue damage and facilitating viral dissemination (27); and enhancing the M6P-dependent cathepsin synthesis to inhibit host antigen presentation and immune phagocytosis (28–30). The other was the host’s antiviral responses and attempts at homeostasis restoration, which were demonstrated by the re-initiation of guanosine triphosphate (GTP) catabolism and purine metabolism during late infection, along with the time-dependent upregulation of proline synthesis and mitochondrial protein synthesis, reflecting the host’s transition from early infection-induced immune exhaustion and metabolic suppression toward tissue repair and metabolic reconstruction (20, 23, 24, 31, 32); the downregulation of fatty acid β-oxidation to support the rapid proliferation of immune cells during early infection, reflecting the host metabolic reprogramming in rapid response to viral infection (15); the lipid metabolism that displayed the coexisting anti-inflammatory and pro-inflammatory factors—the elevated levels of oxidized GSH and ARA indicated enhanced inflammatory responses and aggravated oxidative stress, while the time-dependent increases in the anti-inflammatory mediators (i.e., DTA, DHA, and deoxycholic acid) and antioxidants (e.g., biliverdin and indolelactic acid) reflected the host-mediated homeostatic regulation of inflammation and redox status (33–36); and the time-dependent enhancement of vitamin B6 metabolism that indicated an increased demand for pyridoxal-5'-phosphate (PLP) coenzymes, which correlated with improved immune cell function and inflammatory balance regulation (37).

The metabolic state of KI mice exhibited the following two possible characteristics: one manifested as the loss of dynamic regulation between the pro-inflammatory and anti-inflammatory processes mediated by PUFA/PLA2 (15, 33, 34), which may suggest that the excessive activation of CRP downstream immune events is caused by CRP overexpression and might be associated with the state of immune dysregulation in the host following viral challenge, and the other characterized by an altered Trp metabolism to accommodate the increased demand for immunosuppression and antioxidation, as well as the aberrant alterations in the metabolic checkpoint of innate immunity—myristic acid (38, 39), which demonstrated the occurrence of host countermeasures against CRP overexpression to partially compensate for immune dysregulation.

The metabolic status of KO mice exhibited the following three possible characteristics. Firstly, manifested in the lung tissue as a premature shift of the Trp metabolism toward immunosuppression and a lack of immune-supportive metabolic reprogramming such as the downregulation of the fatty acid β-oxidation-mediated metabolic resource redistribution (15, 25, 40), suggesting that the absence of CRP might be directly associated with unresponsiveness in the downstream CRP-related immune events and the hypoimmune state in the host after viral invasion. Secondly, manifested in the lung tissue as the impaired synthesis of the critical antioxidant substance GSH, the abnormal changes in related metabolite levels, and the impaired synthesis of proline, a key precursor for antioxidant and tissue repair proteins (31, 32, 36, 41), demonstrating that the absence of CRP might be indirectly associated with severe collapse of the host’s antioxidant system and dysfunction in tissue damage repair. Thirdly, manifested in the lung tissue as the increased uptake of indole derivatives from the gut microbiota, upregulation of synthesis or the uptake of multiple amino acids related to energy metabolism or immune function, the hyperactivation of PUFA/PLA2 metabolism, and the early high-intensity upregulation of vitamin B6 uptake and metabolism (33, 34, 37, 38, 42), demonstrating that possible compensatory events occurred in the host against CRP deficiency to partially compensate for the immune, anti-inflammatory, and antioxidative functions.

Notably, in our previous studies, we found that the immune checkpoint LAIR-1 presented significant correlation with viral load in all three types of mice (14). This may be related to LAIR-1 playing a role in the regulation of immune cells and acting as a functional inhibitory receptor to limit respiratory inflammation of airway infiltrating neutrophils (43, 44). In this study, the correlation analysis showed that Kyn presented a significant correlation with LAIR-1, which was positively correlated in WT mice, but negatively correlated in KI and KO mice. In addition, oxidized GSH and Kyn were negatively correlated with viral load in both WT and KI mice. These clues may further underscore the importance of GSH as a key antioxidant and the crucial role of the Kyn-mediated negative immune regulation tendency of Trp metabolism in host antiviral infection (25, 36).

In addition, our study indicated that severe influenza A virus infection altered the gut–lung metabolic axis dynamics, which followed the presented compensatory responses due to CRP deficiency or human CRP transgenic treatment in mice. The gut–lung metabolic axis, a complex bidirectional communication system involving interactions between the gut microbiota, metabolites, immune cells, and signaling molecules across the intestine and the lungs, is mediated by the circulatory transport of soluble microbial components and metabolites (45, 46). Our results presented increased levels of indolelactic acid and deoxycholic acid in the gut microbial Trp metabolism and bile acid metabolism at 7 dpi in WT mice, demonstrating that the gut–lung axis played a role in mitigating the host inflammatory responses and the immune injury during the later stage of infection (35, 47). However, our results also presented the loss of post-infection responsiveness in the gut microbial bile acid metabolism in KI and KO mice. Changes in the 5-HIAA and NMA levels in the Trp metabolism of KI mice at 7 dpi and the sustained increase of the indole derivatives in the Trp metabolism of KO mice indicated compensatory antioxidant support from the gut–lung axis at varying degrees (38, 42).

A notable limitation of this study is that it is entirely correlative, and there is currently a lack of functional experiments to verify the impact of the identified metabolic changes on the disease outcomes after influenza virus infection. A further limitation is the relatively small sample size, which restricts the statistical power and increases the risk of type II errors (false negatives). While our correlative findings provide preliminary insights, the reduced sensitivity of statistical tests in small cohorts may have limited our ability to detect subtle but biologically meaningful associations. Furthermore, small sample sizes are more susceptible to sampling bias, potentially affecting the generalizability of our results. We acknowledge that these constraints necessitate cautious interpretation of the data and underscore the need for replication in larger, independent cohorts to validate our observations. Future studies should prioritize expanding sample sizes to enhance robustness, particularly when exploring multifactorial traits or interactions with moderate effect sizes, and conduct molecular functional validation using cell or animal models, or more precise targeted metabolomics studies, in order to gain a deeper understanding of the relationship between CRP and immune balance in influenza virus infection.

In conclusion, this study described the temporal metabolic change patterns in the lung tissues of mice after severe influenza A virus infection and explored the effect of CRP on severe influenza from the perspective of metabolomics. Our results suggest that deficiency of CRP or human CRP transgenic treatment causes host metabolic reprogramming after influenza infection. The changes in metabolites may represent disease progression in severe influenza. These findings contribute to the understanding of the underlying mechanisms of pathogen–host interactions and may enable the metabolic pathways and molecules characterized in this research to serve as disease progression biomarkers for influenza or as disease-relevant targets for the design of pathophysiology-driven therapeutics to alleviate severe influenza.

## Data Availability

The original contributions presented in the study are included in the article/**Supplementary Material**. Further inquiries can be directed to the corresponding author.
