# Supplementary figures and images for: Metabolic reprogramming induced by CRP deficiency or human CRP transgenic in influenza-infected mice
Source: Front Immunol. 2026 Mar 9;17:1683431. doi: 10.3389/fimmu.2026.1683431 (PMC13006326; doi:10.3389/fimmu.2026.1683431)

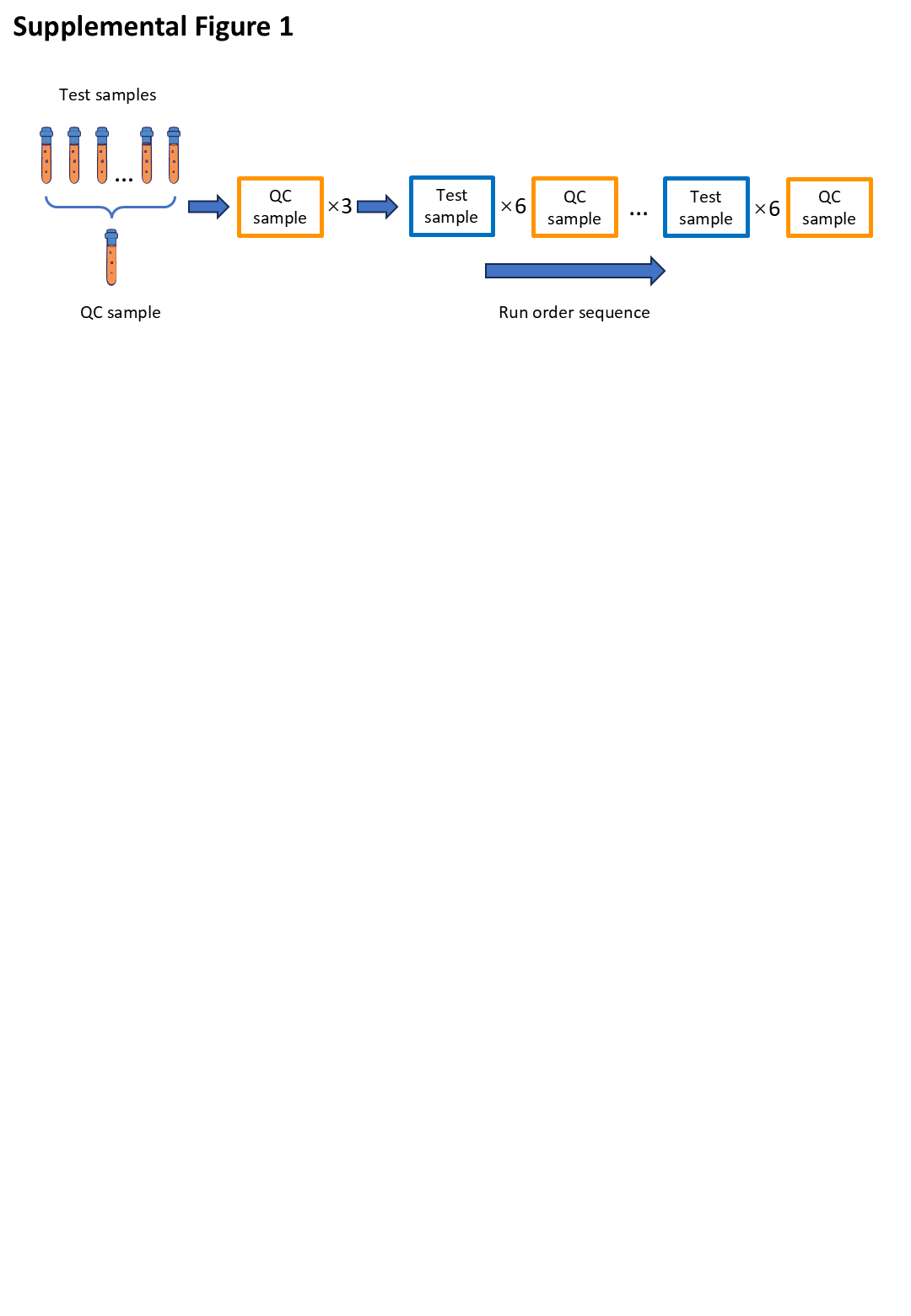

Supplement: Supplementary file 1 [file Image1.tiff]

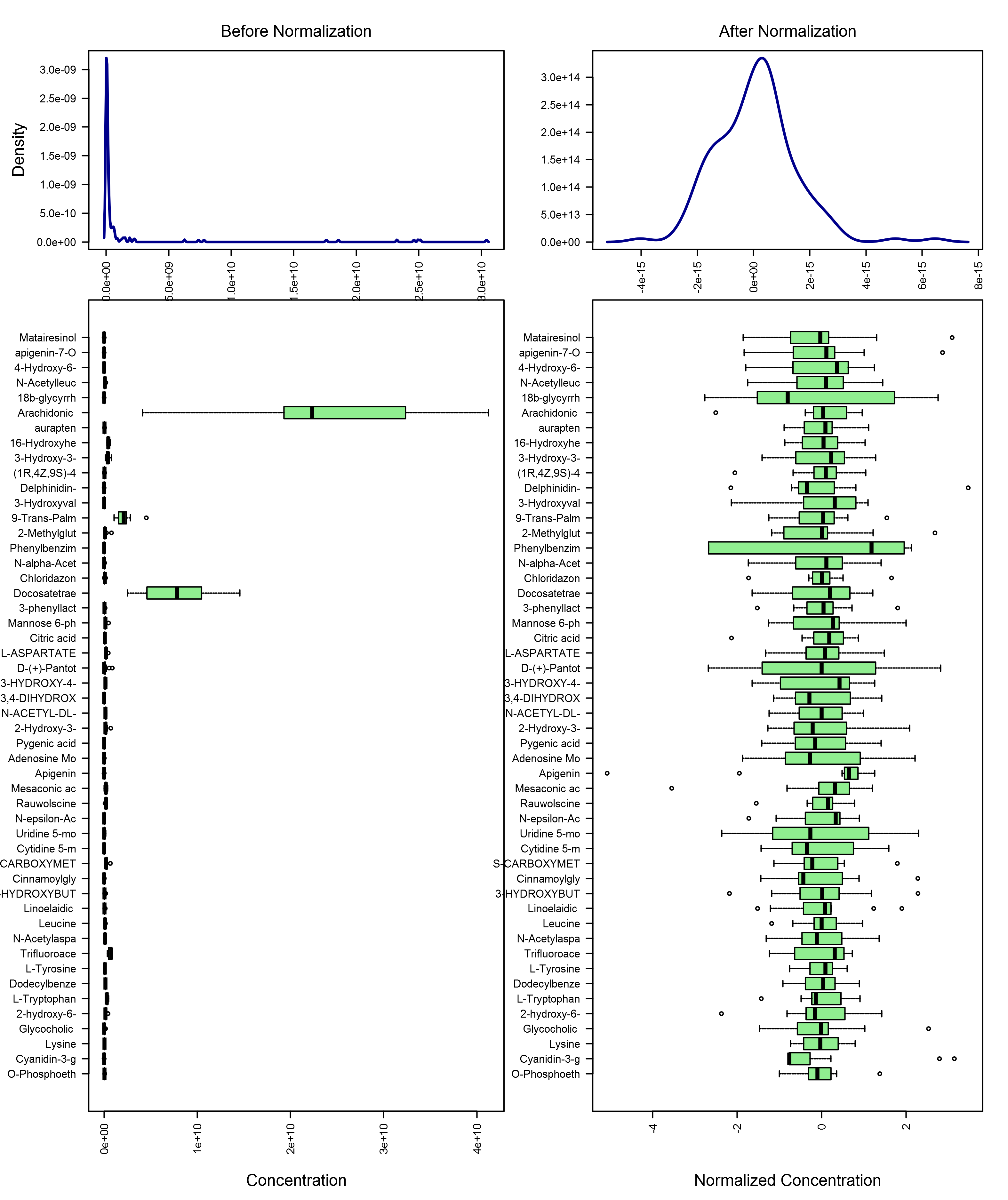

Supplement: Supplementary file 2 [file Image2.png]

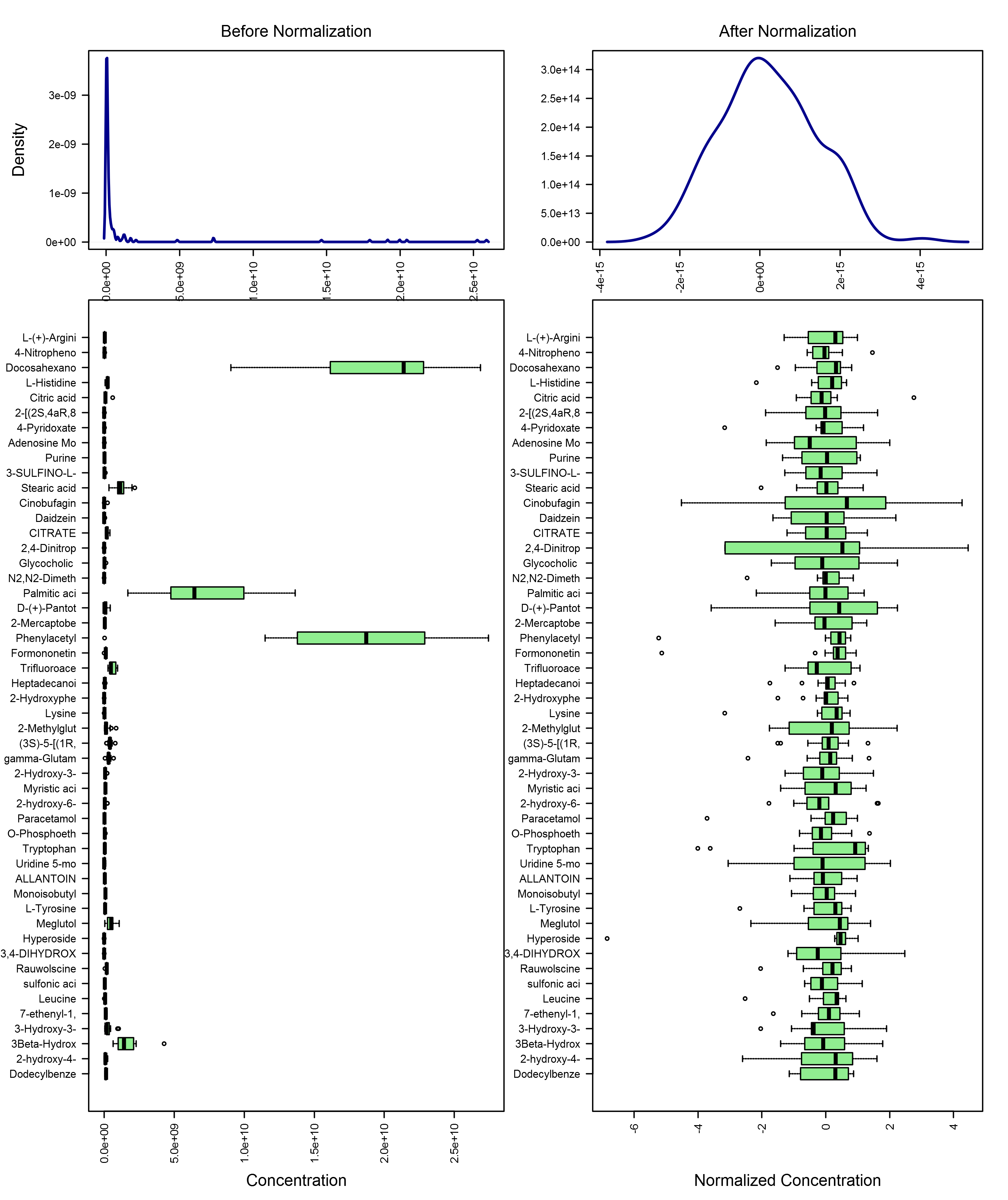

Supplement: Supplementary file 4 [file Image4.png]

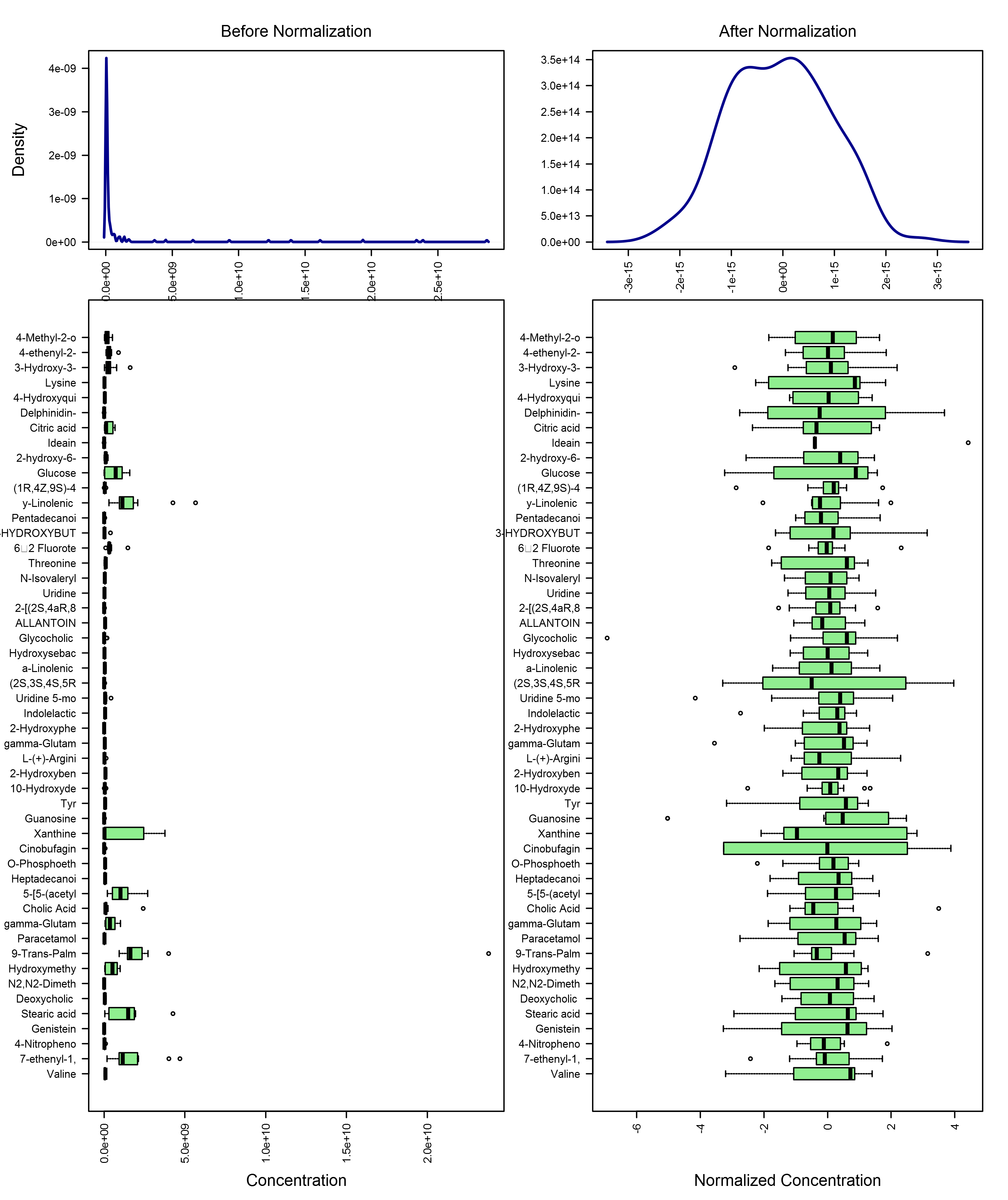

Supplement: Supplementary file 6 [file Image6.png]

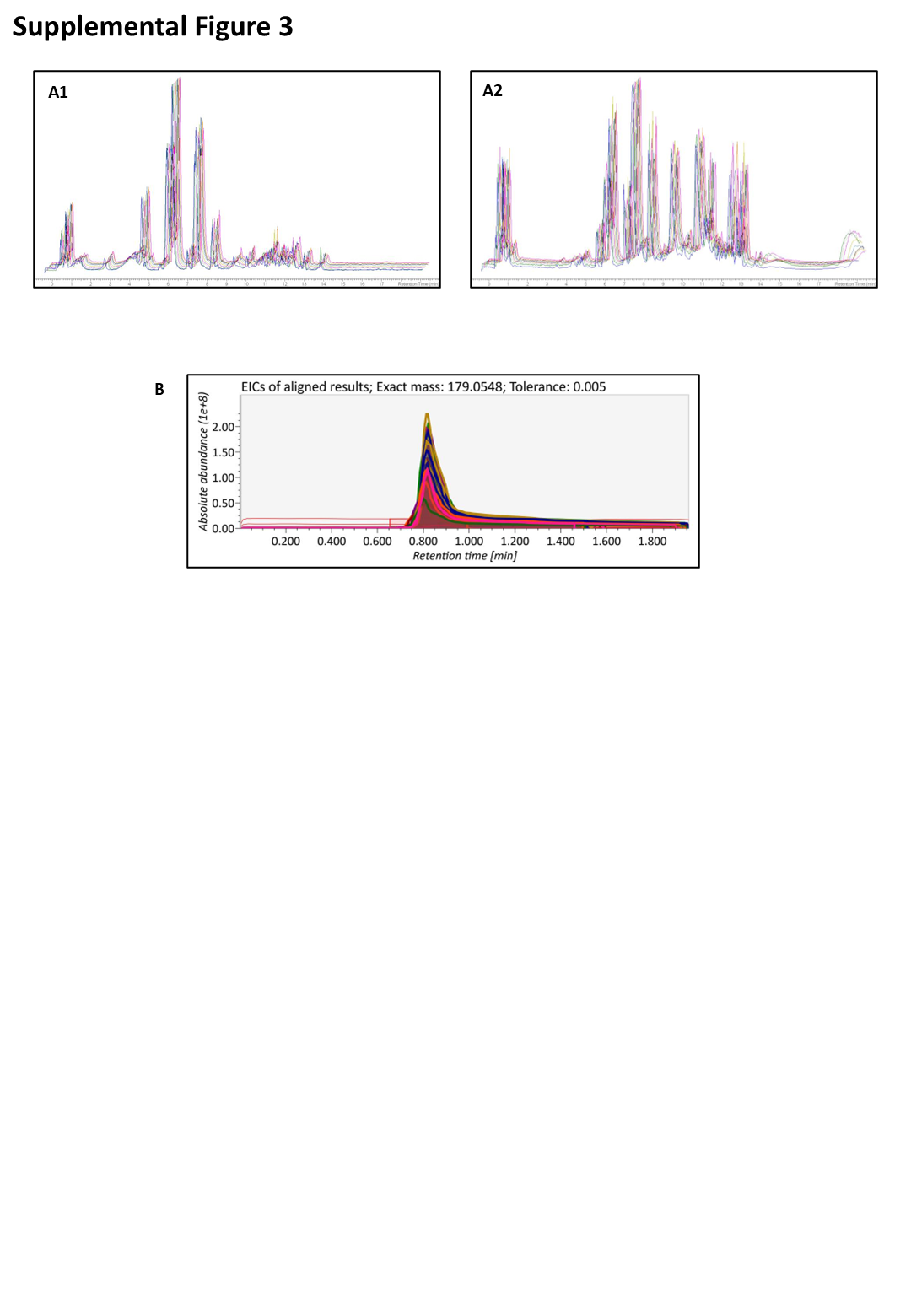

Supplement: Supplementary file 8 [file Image8.tiff]

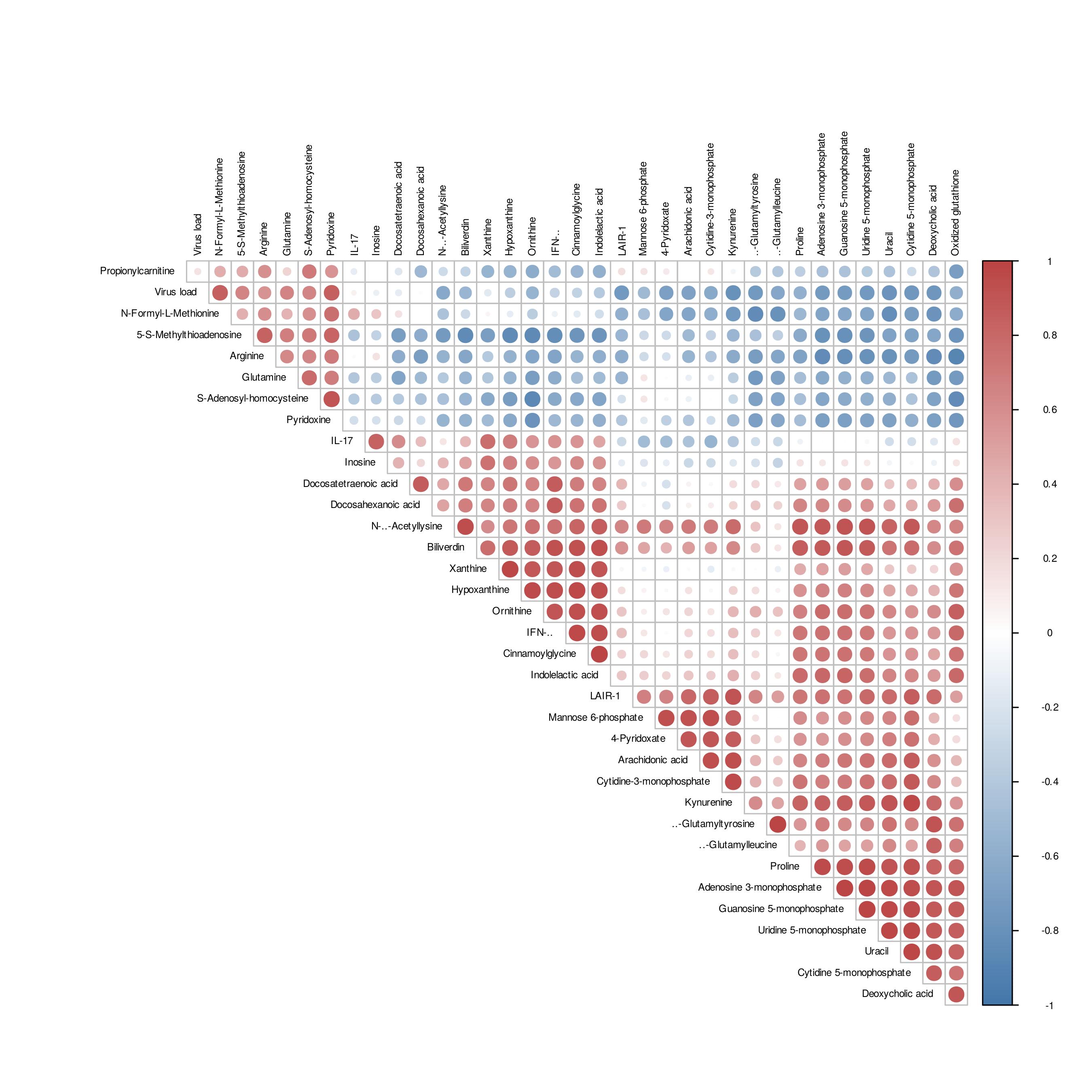

Supplement: Supplementary file 9 [file Image9.jpeg]

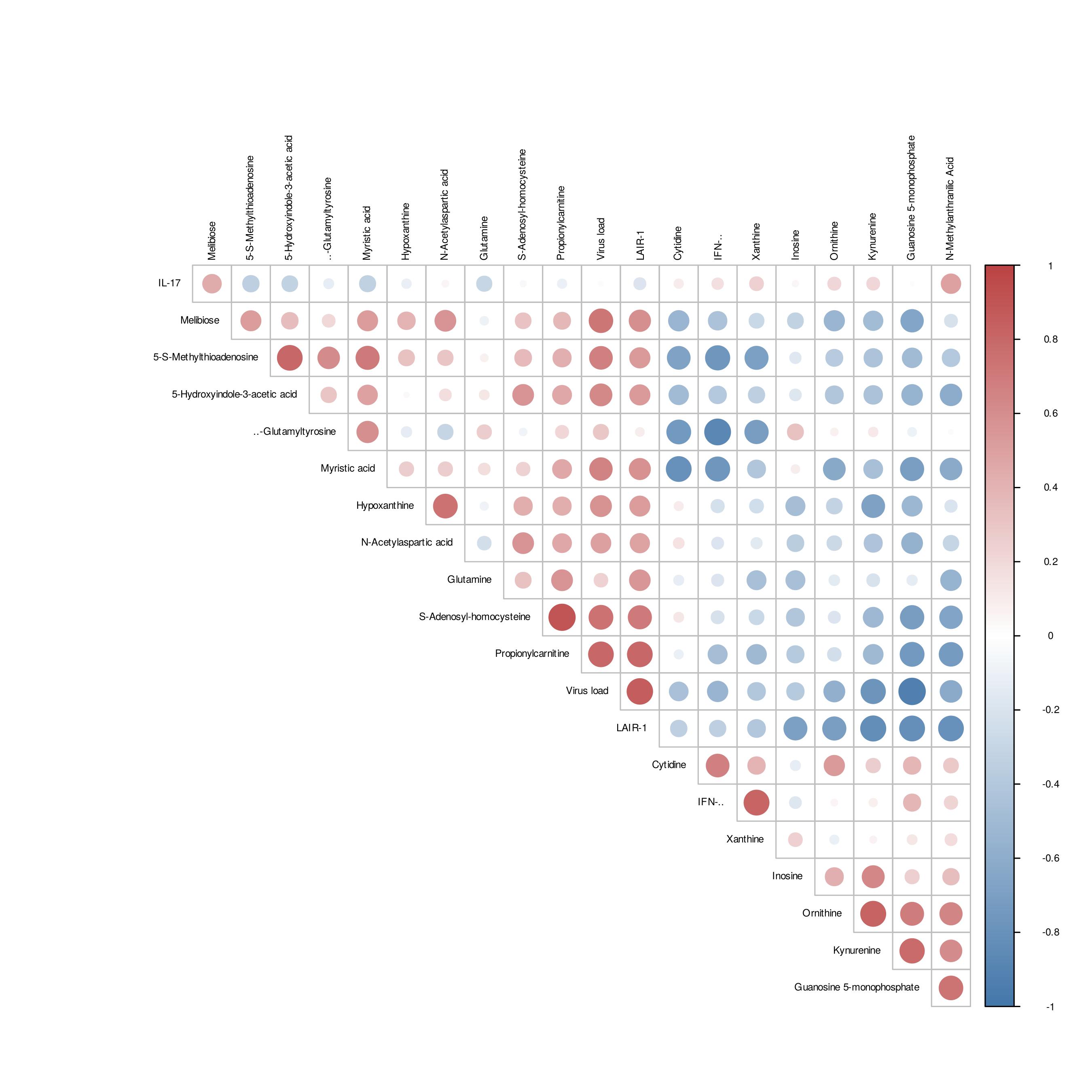

Supplement: Supplementary file 10 [file Image10.jpeg]

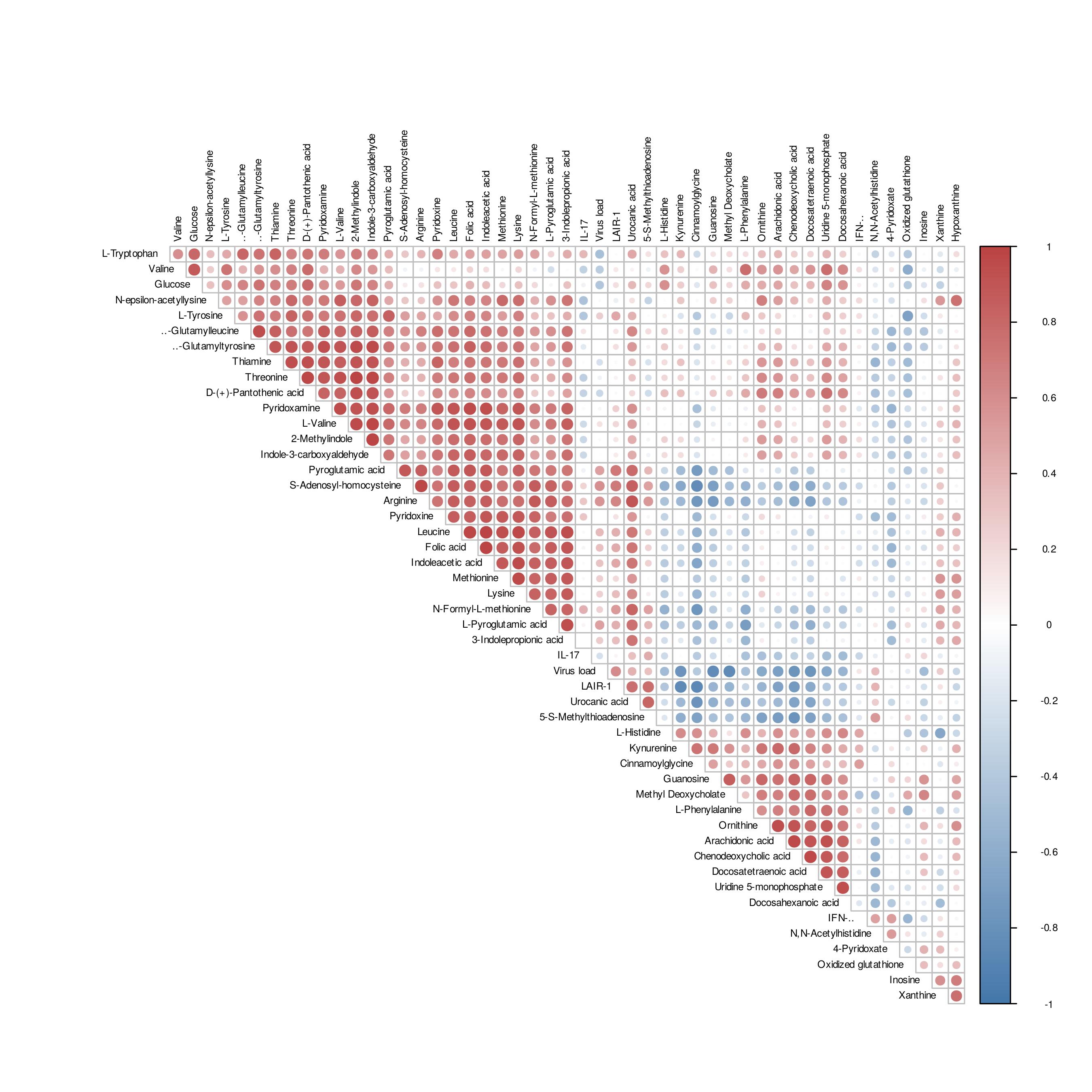

Supplement: Supplementary file 11 [file Image11.jpeg]
