# Supplementary material for: Metabolic reprogramming induced by CRP deficiency or human CRP transgenic in influenza-infected mice
Source: Front Immunol. 2026 Mar 9;17:1683431. doi: 10.3389/fimmu.2026.1683431 (PMC13006326; doi:10.3389/fimmu.2026.1683431)
Supplement: Supplementary file 12 [file Table1.docx]

**Supplemental Table 1**. Chromatographic gradient

| Time (min) | Aqueous phase (liquid A) | Organic phase (liquid B) |
| --- | --- | --- |
| 0 | 90% | 10% |
| 1 | 90% | 10% |
| 13 | 2% | 98% |
| 18 | 2% | 98% |
| 18.5 | 90% | 10% |
| 20 | 90% | 10% |
